# Nitrous oxide‐related neurological disorders: Clinical, laboratory, neuroimaging, and electrophysiological findings

**DOI:** 10.1002/brb3.2402

**Published:** 2021-11-10

**Authors:** Jiwei Jiang, Xiuli Shang, Xiaoting Wang, Hanze Chen, Wenyi Li, Yanli Wang, Jun Xu

**Affiliations:** ^1^ Department of Neurology, Beijing Tiantan Hospital Capital Medical University Beijing China; ^2^ Department of Neurology, The First Affiliated Hospital China Medical University Shenyang China; ^3^ Department of Neurology, Sir Run Run Shaw Hospital, School of Medicine Zhejiang University Hangzhou China

**Keywords:** homocysteine, magnetic resonance imaging, nitrous oxide, subacute combined degeneration, vitamin B_12_ deficiency

## Abstract

**Background:**

Recreational N_2_O abuse is an important etiology of neurological impairment in young patients, which may easily be ignored clinically. Few current studies have investigated the characteristics or the effects experienced by its users. We aimed to explore any correlation between the clinical severity and biomarkers and spinal magnetic resonance imaging (MRI) abnormalities, identify independent factors associated with spinal MRI abnormalities, and ascertain factors affecting depression/anxiety in patients with N_2_O‐related neurological disorders.

**Methods:**

Patients with N_2_O‐related neurological disorders were enrolled retrospectively between February 2017 and July 2020. Their demographic, clinical, laboratory, neuroimaging, electrophysiological, and neuropsychological findings were analyzed. Correlation analyses were conducted using Spearman's or Pearson's correlation and linear regression analysis. Independent factors associated with spinal MRI abnormalities were identified using univariate and multivariate analyses.

**Results:**

The principal clinical manifestations of N_2_O‐related neurological disorders (*n* = 63; 38 men, 25 women; mean age ± SD: 22.60 ± 4.46 years) were sensory disturbance, followed by gait disturbance and pyramidal tract damage. A significant negative correlation existed between serum vitamin B_12_ levels and clinical severity (*r* = −0.309, *p *= .014), which disappeared after linear regression. An interval of less than 6 months between initial N_2_O abuse and hospitalization was independently associated with spinal MRI abnormalities (39.47% vs. 72.00%, respectively; *χ* ^2 ^= 6.40, *p *= .01). Thirty‐eight (60.32%) and 40 (63.49%) patients experienced anxiety and depression, respectively. Moreover, the higher the clinical scores/serum homocysteine levels, the greater the severity of anxiety/depression (*r* = 0.442, *p *< .01; *r* = 0.346, *p *< .01; *r* = 0.477, *p *< .01; *r* = 0.324, *p *< .01).

**Conclusions:**

The significant inverse correlation between initial vitamin B_12_ levels and clinical severity could aid prognosis prediction in patients with N_2_O‐related neurological disorders. Spinal MRI abnormalities were not related to clinical severity but depended on the time interval between initial N_2_O abuse and hospitalization. Anxiety and depression were common comorbidity in these patients, and their severity increased with the intensity of clinical impairment and/or serum homocysteine levels.

## Graphical abstract

We develop a research to explore any correlation between clinical severity and biomarkers and spinal magnetic resonance imaging (MRI) abnormalities, identify independent factors associated with spinal‐MRI abnormalities, and ascertain factors affecting depression/anxiety in patients with N2O‐related neurological disorders.

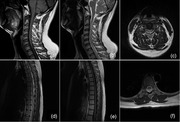

## 1 INTRODUCTION

Nitrous oxide (N_2_O), also known as laughing gas, is a stable, colorless, and sweet‐tasting gas. Historically, N_2_O has played a very important role in providing general anesthesia in clinical settings, including dentistry, ambulance transport, and childbirth (Lew et al., 2018). Recent studies have provided evidence of a sharp surge in N_2_O abuse for recreational purposes, especially among adolescents, owing to its euphoric effect, easy availability, and low cost (Bethmont et al., 2019; Oussalah et al., 2019). Epidemiological studies showed that the frequency of N_2_O abuse rose from 4% of respondents in 2013 to 16% in 2017 (Entwistle & Burns, 2013; O'Donnell & Uporova, 2017). A New South Wales survey on participants’ exposure to stimulants found that 75% of respondents recently used N_2_O in 2018, which increased from 20% in 2013 (Winstock et al., 2017). Accordingly, there have been sporadic reports of the risk of subacute combined degeneration (SCD), peripheral neuropathy, and neuropsychiatric disturbances resulting from exposure to N_2_O (Garakani et al., 2016; Hew et al., 2018; Lan et al., 2019; Yuan et al., 2017).

Despite the increase in its abuse among the youth and reports of N_2_O‐induced neurological complications, current studies remain limited by extremely small sample sizes and incomplete exploration of the clinical severity, biomarkers, neuroimaging, and neuropsychology in these patients (Bao et al., 2020; Zheng et al., 2020). These shortcomings account for the insufficient consideration of N_2_O abuse as an important etiology of polyneuropathy and SCD in young patients, leading to inadequate diagnosis and treatment. Therefore, we conducted a retrospective study to analyze any correlation between the relevant biomarkers, extent of spinal magnetic resonance imaging (MRI) abnormalities, Hamilton Depression/Anxiety (HAMD/HAMA) Rating Scale, and clinical severity in patients with N_2_O‐related neurological disorders, and to identify factors affecting their spinal MRI presentations.

## 2 METHODS

### 2.1 Participants and baseline assessment

This retrospective study enrolled patients with N_2_O‐related neurological disorders who were admitted to the Department of Neurology of the First Affiliated Hospital of China Medical University between February 2017 and July 2020. The participants’ demographic, clinical, laboratory, neuroimaging, electrophysiological, and neuropsychological data from the initial hospitalization were collected. The final diagnoses of N_2_O‐related neurological disorders were established by two experienced neurologists after comprehensive consideration of the clinical symptoms/signs, laboratory data, and radiological and electrophysiological findings. This study was approved by the Ethics Committee of the First Affiliated Hospital of China Medical University and conducted in accordance with the Declaration of Helsinki. All participants provided written informed consent.

### 2.2 Demographic and clinical evaluation

The patients’ demographic data, including sex, age of onset, history of N_2_O abuse, routes of self‐administration, approximate amount used per session, intake frequency (number of time(s)/week), and time interval between first N_2_O use and hospitalization were collated. Two experienced neurologists independently determined the severity of neurological impairment based on the clinical scoring system described by Hemmer et al., which has been widely used by other researchers (Hemmer et al., 1998; Jain et al., 2014). The scoring system comprises five items: gait disturbance (0 = normal, 1 = positive Romberg's sign, 2 = impaired ambulation but able to walk unsupported, 3 = substantial support required for ambulation, and 4 = wheelchair‐bound or bedridden), sensory disturbances (hypesthesia, dysesthesia, vibration/joint‐position impairment; 0 = normal, 1 = impairment in toes and fingers, 2 = impairment in the ankle and wrists, and 3 = impairment in the upper arms and legs), mental impairment (0 = normal, 1 = intellectual or behavioral impairment without the need for social support, 2 = partial dependence for activities of daily living, and 3 = complete dependence for all activities of daily living), neuropathy (0 = no loss or reduction of reflex, 1 = areflexic or hyporeflexic ankle jerk, 2 = areflexic or hyporeflexic patellar jerk, and 3 = areflexic or hyporeflexic biceps jerk), and pyramidal tract signs (0 = no involvement of the pyramidal tract, 1 = positive Babinski sign, 2 = spastic paraparesis, and 3 = spastic tetraparesis). The overall score ranges from 0 to 16. The higher the clinical score, the greater the severity of neurological impairment.

### 2.3 Laboratory and conventional MRI assessments

All patients underwent laboratory tests that assessed their complete blood cell count, vitamin B_12_, folate, and homocysteine levels. Hepatic, renal, and thyroid function; ion levels; blood sugar; antibodies related to rheumatism, paraneoplastic syndrome and autoimmune encephalitis; syphilis; HIV infection, and cerebrospinal fluid were analyzed as needed for the differential diagnosis.

All patients underwent conventional spinal MRI using 1.5‐T/3.0‐T magnetic resonance devices (Magnetom H‐15 and Vision; Siemens, Erlangen, Germany) on admission. The cervical, thoracic, and/or lumbar segments of the spinal cord were scanned, depending on the patient's clinical signs and symptoms, with sagittal and axial reconstruction. Spinal T1‐weighted spin‐echo (repetition time/echo time: 500−550/10−15) and T2‐weighted imaging (3000−4000/100−120) were performed with echo train lengths of 5. Other MRI scan parameters included a 3‐mm section thickness and 1‐mm scanning interval. We recorded the extent and location of the affected spinal cord segments with MRI abnormalities.

### 2.4 Electrophysiological and neuropsychological examinations

Each participant underwent electrophysiological examinations of the median, ulnar, peroneal, tibial, and sural nerves, depending on his/her clinical manifestations. The amplitude, distal latency, and conduction velocity of the compound muscle action potential (CMAP) and amplitude and conduction velocity of the sensory nerve action potential (SNAP) were measured. Neuropsychological assessment was conducted in all patients. The HAMA Scale was used to evaluate the severity of anxiety symptoms, which includes 14 items that are rated from 0 to 4. The severity of depression was evaluated using the HAMD Scale, which consists of 17 items. Items 1, 2, 3, 7, 8, 9, 10, 11, and 15 are rated from 0 to 4, while the other items are rated from 0 to 2. We defined anxiety as a HAMA score of above 6, and depression as a HAMD score of above 7.

### 2.5 Correlation and regression analysis

We explored any correlation between clinical severity and sensitive biomarkers, including serum red blood count (RBC), hemoglobin, mean corpuscular volume (MCV), vitamin B_12_, folate, and homocysteine, and the extent of the spinal MRI abnormalities in patients with N_2_O‐related neurological disorders. Subsequently, we analyzed the correlation and linear regression between clinical severity, serum vitamin B_12_, and homocysteine levels, and the HAMA/HAMD scales in these patients.

### 2.6 Statistical analysis

SPSS 22.0 software (SPSS Inc., Chicago, IL, USA) was used to conduct all statistical analyses. Statistical differences between categorical variables were determined with the *χ* ^2^ or Fisher's exact test and presented as the total numbers (n) and percentages (%) per group. Continuous variables with normal data distribution were analyzed using Student's *t*‐test and presented as means and SDs, while data with non‐normal distribution were analyzed using the Mann–Whitney *U* test and presented as the medians and interquartile ranges (IQRs). Pearson or Spearman correlation (depending on the distribution of the data) and regression analysis were performed to examine the associations between the laboratory, neuroimaging, and neuropsychological variables and clinical severity. Univariate and multivariate analyses were conducted to ascertain the independent factors affecting the spinal MRI manifestations. *p*‐Values ≤.05 were considered statistically significant.

## 3 RESULTS

### 3.1 Patient profiles

Sixty‐three patients with N_2_O‐related neurological disorders (38 men, 25 women) aged between 15 and 33 (mean ± SD: 22.60 ± 4.46) years were enrolled in this study. The patients’ exposure to N_2_O entailed inhalation from bulbs/balloons (7.5 g/10 ml/bulb) or larger canisters (1000 ml/canister); two patients also took N_2_O in the solid form. Accurate quantification of N_2_O inhalation was challenging because users typically inhaled in groups or were unable to describe the precise volumes of the balloons. The amount of N_2_O consumed per session was 4000 (2400−7000) ml (median [IQR]), and the intake frequency was 3.33 ± 1.69 times per week. The time interval between initial N_2_O abuse to hospitalization was 6 (3−12) (median [IQR]) months.

### 3.2 Clinical impairments and severity

Neurological manifestations of the participants are presented in Table 1. Sixty‐one of 63 patients (96.83%) had gait disturbance, and “gait impairment but able to walk unsupported” (27, 42.86%) was the most common presentation. All patients (63, 100%) complained of sensory disturbances, including hypesthesia, dysesthesia, and impaired sense of vibration and position. Moreover, sensory disturbances affected the upper arms and legs bilaterally in most patients (45, 71.43%). Mental disorders were observed in 19 patients (30.15%), among whom “slight impairment without the need for social support” (14, 22.22%) was the most common presentation. Thirty patients (47.62%) with neuropathies showed loss or reduction in the deep tendon reflexes in the patellar and biceps regions. Thirty‐six patients (57.14%) presented with clinical signs of pyramidal tract damage. The clinical scores for all patients ranged from 4 to 13 (mean ± SD: 8.08 ± 2.25).

**TABLE 1:** Scores of neurological impairments in patients with N_2_O‐related neurological disorder

Clinical impairment (*n* = 63)	Number of patients (%)
**Gait disturbance (*n* = 61)**	
Normal	2 (3.27)
positive Romberg's sign	4 (6.35)
impairment but able to walk unsupported	27 (42.86)
substantial support required for ambulation	13 (20.63)
wheelchair or bed bound	17 (26.98)
**Sensory disturbance (*n* = 63)**	
Normal	0
In toes and fingers	6 (9.52)
In ankle and wrists	12 (19.05)
In upper arms and legs	45 (71.43)
**Mental impairment (*n* = 19)**	
Normal	44 (69.84)
Impairment but requires no social support	14 (22.22)
Partially dependent for activities of daily living	4 (6.35)
Completely dependent for all activities of daily living	1 (1.59)
**Neuropathy (*n* = 30)**	
No loss or reduction of reflex	33 (52.38)
Areflexic or hyporeflexic ankle jerk	0
Areflexic or hyporeflexic patellar jerk	17 (26.98)
Areflexic or hyporeflexic biceps jerk	13 (20.63)
**Pyramidal tract signs (*n* = 36)**	
No involvement of the pyramidal tract	27 (42.86)
positive Babinski sign	9 (14.29)
Spastic paraparesis	14 (22.22)
Spastic tetraparesis	13 (20.63)
Clinical scores, mean ± SD (Min–Max)	8.08 ± 2.25 (4–13)

### 3.3 Biomarkers and correlation with clinical severity

The changes in serum levels of RBC, hemoglobin, MCV, vitamin B_12_, folate, and homocysteine of the study population are presented in Table 2. The mean serum RBC level in men and women was (4.51 ± 0.57) ×10^12^/L and (3.94 ± 0.42) ×10^12^/L, respectively. The mean serum hemoglobin level in men and women was 144.43 ± 17.38 g/L and 127.16 ± 10.86 g/L, respectively. The mean serum MCV and folate levels were 95.74 ± 5.27 fl and 18.03 ± 9.92 nmol/L, respectively. The serum vitamin B_12_ level was 229 (107−515) pmol/L (median [IQR]) (maximum measurable value, 1476 pmol/L). The mean serum homocysteine level was 30.95 ± 15.09 μmol/L (maximum measurable value, 50 μmol/L). Elevated homocysteine, which is indicative of functional vitamin B_12_ deficiency at the cellular level, was observed in 55 patients (87.30%). A negative correlation (*r* = −0.309, *p *= .014) was observed only between clinical severity and serum vitamin B_12_ levels; however, no obvious linear dependent relationship was found (*F* = 1.324, *p *= .254). No significant correlation was observed between the clinical scores and serum levels of RBC, hemoglobin, MCV, folate, and homocysteine (*r* = −0.135, *p *= .293; *r* = −0.102, *p *= .427; *r* = 0.121, *p *= .345; *r* = −0.023, *p *= .856; *r* = 0.022, *p *= .866; respectively).

**TABLE 2:** Values and changes in the serum biomarkers

Laboratory data (unit)	Mean ± SD/median (IQR)	Min–Max	Normal range	Data change	Number (%)
RBC (male, ×10^12^/L)	4.51 ± 0.57	2.81–5.45	4.30–5.80	Decrease in RBC	19 (30.16)
RBC (female, ×10^12^/L)	3.94 ± 0.42	3.17–4.66	3.80–5.10		
Hb (male, g/L)	144.43 ± 17.38	89.00–170.00	130.00–175.00	Decrease in Hb	12 (19.05)
Hb (female)	127.16 ± 10.86	107.00–152.00	115.00–150.00		
MCV (fl)	95.74 ± 5.27	85.90–107.30	82,00–100.00	Increase in MCV	14 (22.22)
MCH (pg)	32.65 ± 1.74	28.10–36.40	27.00–34.00		
MCHC (g/L)	340.46 ± 10.59	320.00–364.00	316.00–354.00		
Vitamin B_12_ (pmol/L)	229.00 (107.00−515.00)	60.70–1476.00	145.00–637.00	Increase in vitamin B_12_	13 (20.63)
				Decrease in vitamin B_12_	22 (34.92)
Folate (nmol/L)	18.03 ± 9.92	4.10–45.40	8.83–60.80	Decrease in folate	8 (12.70)
Hcy (μmol/L)	30.95 ± 15.09	5.93–50.00	4.44–13.56	Increase in Hcy	55 (87.30)

Abbreviations: Hb, hemoglobin; Hcy, homocysteine; HIV, human immunodeficiency virus; IQR, interquartile range; MCV, mean corpuscular volume; MCH, mean corpuscular hemoglobin; MCHC, mean corpuscular hemoglobin concentration; Min, minimum value; Max, maximum value; RBC, red blood count.

### 3.4 Conventional MRI findings

**FIGURE 1:**
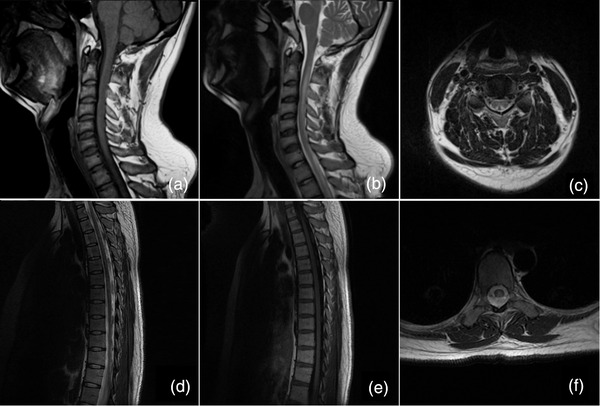
Conventional magnetic resonance imaging of the spinal cord. A 24‐year‐old man presented with impairment but was able to walk unsupported and numbness in all extremities. Sagittal T1‐weighted imaging showed decreased intramedullary signal intensity along the posterior column of the spinal cord extending from C1 to C5 (a) with corresponding hyperintensities on T2‐weighted imaging (b). Axial T2‐weighted imaging at the C4 level showed an inverted V‐shaped hyperintensity (c). A 23‐year‐old woman in a wheel‐chair presented with numbness in all extremities. Sagittal T1‐weighted imaging showed abnormal hypointensities involving the posterior columns of the spinal cord extending from T2 through T4 (d) with corresponding multiple increased intramedullary signal intensities on T2‐weighted imaging (e). An inverted V‐shaped hyperintensity was seen on axial T2‐weighted imaging at the T4 level within the dorsal thoracal spinal cord (f)

All patients underwent conventional spinal MRI, depending on their clinical manifestations and neurological examination. However, only 38 (60.31%) patients presented with typical spinal cord abnormalities, demonstrating the relatively poor sensitivity of MRI for the detection of N_2_O‐induced neurological lesions. The findings included symmetrical hypointensities on T1‐weighted imaging and hyperintensities on T2‐weighted imaging that involved the posterior columns of the cervical or thoracic spine, with an inverted V‐sign on axial MRI (Figure 1), which was consistent with SCD of the spinal cord. The cervical spine (*n* = 37, 58.73%) was most commonly affected in patients with MRI signal abnormalities, often involving the C2 to C6 segments, followed by the thoracic spine (*n* = 7, 11.11%). Longitudinally extensive lesions spanning five or more vertebral segments were observed in 31 patients (49.21%). Lumbar spinal segment abnormalities were not observed in any participant.

Univariate measures showed that the interval between initial N_2_O abuse and hospitalization exceeded 6 months more frequently in patients with normal spinal MRI findings than those with signal abnormalities (72.00% vs. 39.47%; *χ* ^2 ^= 6.40, *p *= .01, Table 3). No differences were observed in the age of onset; sex; serum levels of RBC, hemoglobin, MCV, vitamin B_12_, and homocysteine levels; axonal and/or demyelinating neuropathy on electromyography between patients with normal and abnormal spinal MRI findings (*p *> .05, Table 3). Based on these findings, we included the age of onset, sex, MCV, and interval exceeding 6 months in the multivariate logistic regression model. This analysis showed that initial N_2_O abuse and hospitalization exceeding 6 months [OR = 3.93, 95% confidence interval (CI): 1.26−12.30, *p *= .02] were independently associated with abnormal spinal MRI presentation. Furthermore, we found no significant correlation between clinical severity and extent of spinal involvement in patients with abnormal spinal MRI presentation (*r* = 0.192, *p *= .249).

**TABLE 3:** Values and changes in the serum biomarkers

Variables	Abnormal MRIMean ± SD, median (IQR) or *n* (%)	Normal MRIMean ± SD, median (IQR) or *n* (%)	*t/χ2/Z*	*p*‐Value
**Demographics**				
Age of onset, years	22.16 ± 4.27	23.28 ± 4.76	−0.95	.35
Sex (male)	21 (55.26)	17 (68.00)	1.02	.31
Interval between N_2_O abuse and hospitalization exceeding 6 months	15.00 (39.47)	18 (72.00)	6.40	.01
**Clinical scores**	8.29 ± 2.19	7.76 ± 2.35	0.90	.37
**Laboratory biomarkers**				
RBC (10^12^/L)	4.23 ± 0.56	4.37 ± 0.62	−0.89	.38
Hb (g/L)	136.76 ± 17.01	138.80 ± 17.97	−0.45	.66
MCV (fl)	96.42 ± 5.29	94.69 ± 5.16	1.29	.20
Vitamin B_12_ (pmol/L)	229.75 (107.53–482.00)	216.70 (98.30–538.20)	−0.06	.95
Hcy (μmol/L)	29.80 ± 14.57	32.69 ± 15.98	−0.73	.47
**Electromyography (abnormal)**	31 (81.58)	20 (80.00)	0.02	.88

Abbreviations: Hb, hemoglobin; Hcy, homocysteine; MCV, mean corpuscular volume; MRI, magnetic resonance imaging; RBC, red blood cells.

### 3.5 Electromyographical and neuropsychological examinations

Fifty‐one (80.95%) patients in this study presented with typical mixed axonal and demyelinating neuropathy. Reduced amplitude, prolonged distal latency, and slow conduction velocity of CMAP were observed most frequently in the peroneal and tibial nerves (*n* = 47, 74.60%), while the proportion of patients with reduced amplitude, prolonged distal latency, and slow conduction velocity of SNAP in the median/ulnar nerves and peroneal/tibial nerves was similar (*n* = 32, 50.79% vs. *n* = 34, 53.97%). Thus, the results of the nerve conduction study suggested that atypical sensorimotor neuropathy was common in patients with N_2_O‐related neurological disorders and more prominent in the lower limbs.

**FIGURE 2:**
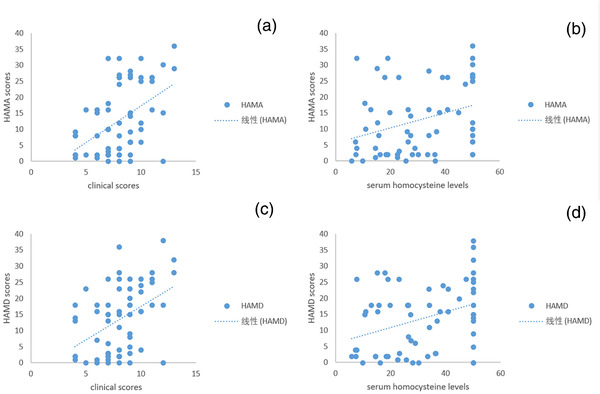
Scatter plot of the correlation and regression between Hamilton Depression/Anxiety (HAMA/HAMD) scores and clinical scores and serum homocysteine levels. A significant positive correlation (*r* = 0.477, *p *< .01) was observed between the clinical severity score and HAMA scores in patients with N_2_O‐related neurological disorders (a). There was a significantly positive correlation (*r* = 0.324, *p *< .01) between the serum homocysteine levels and HAMA scores (b). A significant positive correlation (*r* = 0.442, *p *< .01) was seen between the clinical severity score and HAMD scores in these patients (c). Serum homocysteine levels had significantly positive correlation with HAMD scores (*r* = 0.346, *p *< .01) in our patients (d)

Thirty‐eight (60.32%) patients presented with different degrees of anxiety symptoms, 40 patients (63.49%) presented with different degrees of depressive symptoms, while anxiety and depression coexisted in 38 (60.32%) patients. The analysis of the correlation between the HAMA/HAMD scores and clinical severity, and serum levels of vitamin B_12_, folate, and homocysteine revealed that the HAMA score was positively correlated with clinical severity and serum homocysteine levels (*r* = 0.477, *p *< .01; *r* = 0.324, *p *< .01). The HAMD score was also positively correlated with clinical severity and serum homocysteine levels (*r* = 0.442, *p *< .01; *r* = 0.346, *p *< .01). However, no linear correlation was observed between the HAMA/HAMD scores and serum levels of vitamin B_12_ and folate (*r* = −0.181, *p *= .16; *r* = 0.149 *p *= .24; *r* = −0.189, *p *= .14; *r* = 0.198 *p *= .12). Linear regression analyses were conducted between the HAMA/HAMD scores and the clinical severity and serum homocysteine levels, respectively. Accordingly, the higher the clinical severity or serum homocysteine levels, the greater the severity of anxiety or depression (*F* = 14.85, *p *< .01; *F* = 8.30, *p *< .01; *F* = 18.00, *p *< .01; *F* = 7.18, *p *< .01) (Figure 2).

## 4 DISCUSSION

Recent case studies have reported that N_2_O abuse can result in several neurological and psychiatric disorders, such as SCD, myelopathy, demyelinating polyneuropathy, and even death (Backstrom et al., 2015; Chen et al., 2018; Choi et al., 2019; Edigin et al., 2019; Neveu et al., 2019). Fortunately, these conditions are treatable, and full clinical recovery is also possible if early detection and adequate treatment are implemented (Green et al., 2017; Jain et al., 2014). However, the adverse effects of N_2_O exposure are often ignored clinically, despite its widespread abuse amongst the youth and the surge in the number of reported N_2_O‐induced neurological complications (Kaar et al., 2016). Currently, several case series have focused on the clinical characteristics and biomarkers of N_2_O‐induced neurological damage, whereas few studies have investigated the characteristics of its users or the effects experienced by them. The novel findings of this retrospective study that investigated the above‐mentioned aspects are as follows. First, sensory disturbance was the primary clinical manifestation in patients with N_2_O abuse. Second, a significant negative correlation was observed only between serum vitamin B_12_ levels and severity of neurological impairment, although this relationship was not reflected on linear regression. Third, patients with shorter clinical courses, that is, an interval of less than 6 months between the first N_2_O abuse and hospitalization, were susceptible to spinal cord abnormalities on MRI. Finally, anxiety/depression is more common in these patients than generally believed, and the higher the clinical severity scores or serum homocysteine levels, the greater the degree of anxiety or depression.

Each of the 63 patients included in the present study complained of sensory disturbances, including hypesthesia, dysesthesia, vibration, and impaired proprioception, followed by gait disturbance, which was consistent with previously published studies (Patel et al., 2018; Zheng et al., 2020). Moreover, most instances of sensory disturbance occurred bilaterally in the upper arms and legs. This corresponded to the electromyographical results showing that sensorimotor neuropathy was common in patients with N_2_O‐related neurological disorders and more prominent in the lower extremities. Our findings concurred with the results of Zheng et al. (2020) and Keddie et al. (2018), that is, altered sensation in the extremities was the predominant presenting feature of N_2_O‐related neurological disorders.

Vitamin B_12_ deficiency is a well‐established cause of neurological disorders in elderly individuals with malabsorption syndromes and inadequate intake or bioavailability of vitamin B_12_ (Green et al., 2017), and its incidence generally increases after the age of 60 years (Allen, 2009; Hunt et al., 2014). Clinically, the classical hematological or neurological manifestations of B_12_ deficiency are relatively uncommon because the substantial hepatic stores of vitamin B_12_ can delay clinical manifestations for up to 10 years after the onset of deficiency (Carmel, 2000). This is why patients with vitamin B_12_ deficiency are likely to be elderly individuals. The present study demonstrated that N_2_O‐related neurological disorders were more prevalent among young adults and even adolescents, and the median interval between the first N_2_O abuse and hospitalization was only 6 months, which differs from the established knowledge of patients with conventional B12 deficiency. As reported earlier, recreational N_2_O is legal, inexpensive, and easily available, and young users often inhale N_2_O from shared filled balloons and whipped cream dispensers at house parties or festivals for rapid and transient euphoric effects (Advisory Council on the Misuse of Drugs (ACMD), 2015). Similarly, Nabben et al. (2014) reported that N_2_O use was higher among adolescents aged under 20 years (75%) and young adults in their early twenties (78%) compared to other age groups. The U.S. National Poison Data System also reported that N_2_O use was the highest among adolescents aged 12–17 years (Marsolek et al., 2010). The shift in the affected population suggests that clinicians should consider recreational N_2_O use in young patients who present with neurological symptoms resembling vitamin B_12_ deficiency.

N_2_O can irreversibly inactivate vitamin B_12_ (cobalamin) by oxidizing the cobalt I ion (Co^+^) to Co^2+^/Co^3+^, which consequently leads to functional vitamin B_12_ deficiency (Hathout & El‐Saden, 2011), resulting in two significant dysfunctions. First, the oxidized cobalt cation inhibits the action of cobalamin, a coenzyme for methionine synthase, and further impairs the generation of tetrahydrofolate (THF) and methionine from homocysteine and 5‐methyl‐tetrahydrofolate (5‐methyl‐THF) (Green et al., 2017; Hunt et al., 2014). THF is the precursor to thymidine monophosphate (TMP) required for DNA synthesis. Therefore, the failure of this pathway can result in megaloblastic anemia due to the interference in DNA synthesis and blood cell turnover (Nunn, 1987). Second, vitamin B_12_ acts as a cofactor in the conversion of methylmalonyl‐CoA to succinyl‐CoA (Green et al., 2017; Hunt et al., 2014). Methylmalonyl‐CoA aggregation occurs due to vitamin B_12_ deficiency, which in turn results in methylmalonic acid (MMA) accumulation, thus disrupting lipid synthesis and causing neuronal demyelination (Richardson, 2010). Hence, elevated serum levels of homocysteine and/or MMA serve as better biomarkers than decreased serum vitamin B_12_ for the precise and timely detection of cellular vitamin B_12_ deficiency, consistent with the findings of previous studies (Bao et al., 2020; Buizert et al., 2017; Lan et al., 2019). However, we did not find any linear correlation between the serum homocysteine levels and severity of impairment. On the contrary, a significant negative correlation was observed between the initial serum vitamin B_12_ level and clinical severity. Although this relationship was not reflected on linear regression, this correlation has not been described before in patients with N_2_O‐related neurological disorders, which suggests that serum vitamin B_12_ levels could aid in the prediction of the severity of clinical impairment and even prognosis.

In this study, MRI depicted spinal cord abnormalities in 38 (60.31%) patients, with relatively low sensitivity. Symmetrical T2‐signal abnormalities involving the posterior column of the spinal cord with an inverted V‐sign were predominant and relatively specific manifestations in these patients, consistent with previous studies (Bao et al., 2020; Johnson et al., 2018; Keddie et al., 2018; Patel et al., 2018), which tended to involve the cervical or cervico‐thoracic spinal cord (Lim, 2011; Zheng et al., 2020). This is also in accordance with the characteristics of SCD of the spinal cord, that is, swelling of the myelin sheath and patchy myelopathic spongy vacuolation (Powers, 1996). As noted above, vitamin B_12_ is essential for the maintenance of the myelin sheath; thus, N_2_O‐induced vitamin B_12_ deficiency can account for the progressive demyelination and even axonal loss. Moreover, the highest density of myelinated fibers in the fasciculus gracilis in the cervical area can explain the susceptibility of the cervical spinal cord to N_2_O neurotoxicity (Ohnishi et al., 1976). Interestingly, our study also found that patients with an interval of less than 6 months between the first N_2_O abuse and hospitalization were more likely to present with abnormal signals on spinal cord MRI, in agreement with the findings of Cao et al. (2018). The definitive pathophysiology of the N_2_O‐induced alterations in the spinal cord remains unclear. Some studies demonstrated that intramyelin edema of the spinal cord white matter was responsible for the classical hyperintensities on T2‐weighted imaging in the acute stage (Briani et al., 2013), and stable gliosis replaced cytotoxic edema in the chronic stage of SCD (Murata et al., 1994). Thus, clinicians should be aware of the limitations of conventional spinal MRI in assessing patients with N_2_O‐related neurological disorders because they are not necessarily commensurate with the clinical symptoms and abnormal serum biomarkers.

Our findings revealed that nearly 40 patients had different degrees of anxious and/or depressive symptoms, and that anxiety/depression was a common comorbidity. Furthermore, the degree of anxiety/depression increased with the severity of clinical impairment and/or serum homocysteine levels. Although several studies have demonstrated the positive correlation between anxiety/depression severity and homocysteine (Chung et al., 2017; Esnafoglu & Ozturan, 2020), this relationship has not been examined in patients with N_2_O‐related neurological disorders. Homocysteine‐related methylation reactions are crucial for the health and function of brain tissue. Increased homocysteine can reduce the synthesis of catecholaminergic and non‐catecholaminergic neurotransmitters related to S‐adenosylmethionine that contribute to depression (Zaric et al., 2019). Excess homocysteine also leads to the production of homocysteic acid and cysteine sulfinic acid, which exert excitotoxic effects on the N‐methyl‐D‐aspartate receptor and neurotoxic effects on dopaminergic neurons (Bhatia & Singh, 2015). Current research on the relationship between anxiety and homocysteine is sparse compared to that between homocysteine and depression (Coplan et al., 2015). One possible mechanism underlying the link between homocysteine and anxiety may be the oxidative status of the brain. A recent animal study indicated that hyperhomocysteinemia induced by dietary overload of methionine increases anxiety‐related behaviors in rats, and the proanxiogenic effects could have resulted from oxidative stress in the rat brain (Hrncic et al., 2016).

Our study has some limitations. First, this single‐center retrospective study used consecutively collected data. Therefore, our findings may not be applicable to other settings due to the inherent selection bias. Second, it was difficult to identify the exact amount of N_2_O abused because patients usually inhale it in a group, or they cannot describe the precise volumes contained within the balloons. Thus, we could not analyze the relationship between the inhalation dose and clinical severity and MRI/laboratory findings. Finally, MMA evaluation was not conducted in this study, and concurrent drug/toxin/vitamin‐related diseases were also not incorporated in the analysis.

## 5 CONCLUSION

Recreational N_2_O abuse that resembles the symptoms of vitamin B_12_ deficiency is more common in young and otherwise healthy patients than previously believed. The negative correlation between serum vitamin B_12_ levels with clinical severity suggests that initial serum vitamin B_12_ level may help in determining the prognosis of these patients. Conventional spinal MRI may have relatively low sensitivity in detecting the N_2_O‐related neurological disorders and the presence of spinal lesions is affected by the course of the disease. Since the degree of anxiety/depression increased with the severity of clinical impairment and/or serum homocysteine levels, neurologists are encouraged to screen patients with N_2_O‐related neurological disorders for comorbid anxiety/depression.

## CONFLICT OF INTEREST

The authors declare no conflict of interest.

## AUTHOR CONTRIBUTIONS

*Design of the study, interpretation of data, drafting and revising the manuscript*: Jiwei Jiang. *Conceptualization and critical revision of the manuscript*: Xiuli Shang. *Data collection and ethics submission*: Xiaoting Wang. *Data collection and analyses*: Hanze Chen. *Data analyses*: Wenyi Li and Yanli Wang. *Critical revision of the manuscript, study supervision and funding support*: Jun Xu.

### PEER REVIEW

The peer review history for this article is available at https://publons.com/publon/10.1002/brb3.2402

## Data Availability

The anonymized data that support the findings of this study are available from the corresponding authors upon reasonable request.
